# Treatment of hypothyroidism using Korean medicine: 2 case reports

**DOI:** 10.1097/MD.0000000000019737

**Published:** 2020-05-01

**Authors:** Hyongjun Kim, Sun-Young Moon, Kyungsun Han, Jun-Hwan Lee, Jong Hwan Im, Sungha Kim, Jeong-Eun Yoo

**Affiliations:** a Chun-Jin Korean Medicine Clinic, 89 Jungang-ro, Boryeong-si, Chungcheongnam-do, Republic of Korea; b Clinical Medicine Division, Korea Institute of Oriental Medicine; c Korean Medicine Life Science, University of Science and Technology, Daejeon; d Yakson Korean Medicine Clinic, Taean-gun, Chungcheongnam-do; e Department of Obstetrics and Gynecology, College of Korean Medicine; f Department of Obstetrics and Gynecology, Dunsan Korean Medicine Hospital of Daejeon University, Daejeon, Republic of Korea.

**Keywords:** acupuncture, case reports, hypothyroidism, Korean medicine, thyroid function test

## Abstract

**Introduction:**

Hypothyroidism, the most common endocrine disease, comprises a deficiency of thyroid hormone, causing coldness, fatigue, and dysmenorrhea. Here, we report the improvement of hypothyroidism symptoms and thyroid hormone level normalization by using Korean herbal medicine and acupuncture therapy.

**Patient concerns:**

A 30-year-old woman (Case 1) presented at the clinic with continuous seborrheic dermatitis on the scalp, accompanied by dysmenorrhea. A 55-year-old woman (Case 2) presented with symptoms of coldness of the limbs and fatigue.

**Diagnosis:**

Both patients were diagnosed with “Yin deficiency and Yang hyperactivity” and hypothyroidism after serum thyroid function tests.

**Interventions:**

Both patients received herbal medicine decoction, acupuncture, and electroacupuncture therapy.

**Outcomes:**

Korean medicine improved the symptoms of hypothyroidism and significantly normalized thyroid-stimulating hormone and free-thyroxine levels.

**Conclusion:**

These outcomes suggest that Korean medicine may be effective for resolution of hypothyroidism; however, further research is needed to confirm these findings.

## 1 Introduction

Hypothyroidism, the most common endocrine disorder, is caused by malfunction of the thyroid gland, which produces thyroid hormone.^[1]^ Hypothyroidism is primarily diagnosed by thyroid function test, which measures the amounts of thyroid-stimulating hormone (TSH), free-thyroxine (fT4), and triiodothyronine (T3) in whole blood.^[2]^ The prevalence of hypothyroidism is 4–15% worldwide; in Korea, hypothyroidism affects 14% of the total population, which is a relatively large proportion.^[3–5]^ Primary symptoms of hypothyroidism include dry skin, increased sensitivity to cold, chronic fatigue, muscle cramps, constipation, and hoarseness. Dysmenorrhea can also occur.^[2]^

Levothyroxine supplementation is the standard and primary method for treatment of hypothyroidism. Hypothyroidism patients must maintain a precise dosage of levothyroxine to prevent side effects, such as nausea, vomiting, diarrhea, headache, and muscle cramps.^[6,7]^ Although most patients comply with levothyroxine drug therapy and the level of TSH is generally normalized,^[8]^ a subset of symptoms that accompany hypothyroidism, such as neurologic disorders, are not entirely eliminated.^[9,10]^ Furthermore, it remains controversial whether patients with subclinical hypothyroidism should be administered levothyroxine. The levothyroxine administration adherence rate has decreased in proportion to the treatment duration.^[11]^ Novel treatments are required to appropriately manage overall health care for affected patients.

Complementary and alternative medicine (CAM) also plays a role in the treatment of hypothyroidism, as it enhances patients’ quality of life. Case studies have been published regarding the application of acupuncture in patients with hypothyroidism; they have shown effectiveness in treatment of thyroid dysfunction.^[12–14]^ Herbal medicines have been applied to patients with hypothyroidism, and have been successful for improvement of edema, nocturnal enuresis, fatigue, and anorexia.^[15]^ However, there have been no clinical trials regarding the effects of CAM (eg, herbal medicine and acupuncture) on hypothyroidism and thyroid hormone release. Therefore, we report 2 cases in which hypothyroidism symptoms were alleviated by using Korean medicine, as evaluated by thyroid function test.

## 2 Case presentation

This report describes 2 patients with hypothyroidism who were treated at the Chun-Jin Korean Medicine Clinic (South Korea). The Institutional Review Board of the Korean Institute of Oriental Medicine approved this study (KIOM I-1710/002-001), and informed written consent was obtained from both patients for publication of this case report and accompanying images.

### 2.1 Case 1

A 30-year-old woman visited the clinic with the complaint of continuous seborrheic dermatitis on scalp with symptoms of itching (visual analogue scale, VAS 10), erythema, and scaliness, despite sporadic usage of steroid drug and ointments for 10 years. In addition, hair line was reddish red. She also experienced dysmenorrhea with black menstrual blood. Her menstrual period had been present once every 2 to 3 months since 2008, following headache and low back pain. The patient reported VAS rating of 10 for both seborrheic dermatitis and menstrual pain.

A Korean medicine doctor with 15 years of experience diagnosed this patient with “Yin deficiency and Yang hyperactivity," a type of pattern identified in Korean medicine, based on the symptoms including a feeling of heat, sweating on the face, insomnia, dysmenorrhea, and coldness of lower extremities.^[16]^

To evaluate thyroid function, serum TSH and fT4 levels were measured from baseline to the end of treatment using a blood analysis device (I-Chroma, Biotechmed Inc., Chungcheon-si, Gang-won-do, Republic of Korea). Primary hypothyroidism was diagnosed based on TSH and fT4 levels of 76.18 μIU/mL and 63.8 nmol/L, respectively.

**Figure 1:**
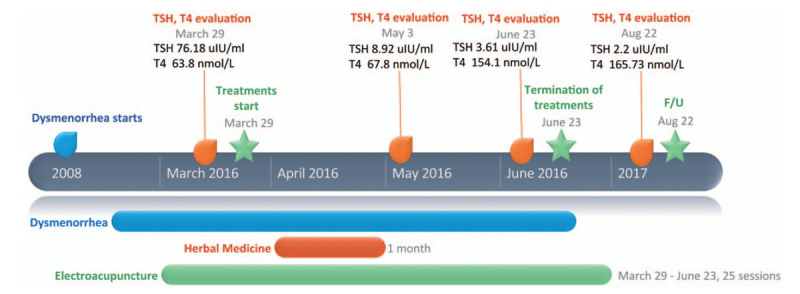
Flow chart depicting treatment in Case 1.

The patient underwent treatment with acupuncture and herbal medicine, based on the Yin deficiency and Yang hyperactivity pattern (Fig. 1). The herbs were administered 3 times per day for 1 month. Acupuncture needles (0.25 mm diameter, 30 mm length; Woojin Acupuncture Corporation, Seoul, South Korea) stimulated the following points: CV17, EX2, CV12, CV4, LI4, LV3, and ST36. Electroacupuncture was also used to stimulate those same acupoints at 1 to 2 Hz for 10 minutes per session (STN-330, StraTek Co., Ltd., Anyang, Republic of Korea). The composition and dosage of herbal medicine decoction are shown in Table 1. Acupuncture was applied 25 times over a period of 4 months.

**Table 1:**
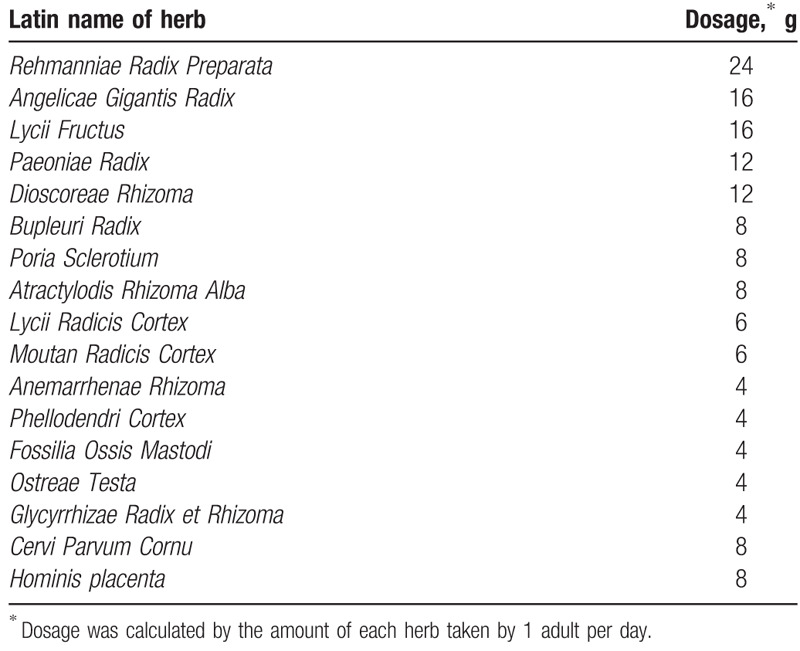
Composition and dosage of herbal medicine used in this report.

**Figure 2:**
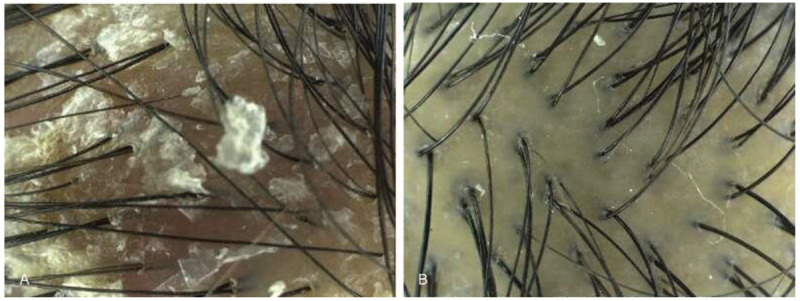
Reduction of scaly scalp after treatment: (A) before treatment (March 29, 2016) and (B) after treatment (June 23, 2016) in Case 1.

After 1 month of treatment, the patient's scalp color changed from dark red to white, despite residual scales (Fig. 2). The dysmenorrhea was improved, whereas the yellowish vagina discharge remained at the time of the third visit in June 2016. After 1 month of herbal medicine administration and 25 acupuncture treatments, the scalp itchiness had nearly vanished, and only appeared upon alcohol consumption. The patient's menstrual cycle became consistently 29 days in length and no further blood clots were released. Her VAS rating for menstrual pain decreased from 10 to 2; her VAS rating for scalp itchiness decreased from 10 to 3 after treatment and scaly scalp was obviously reduced (Fig. 2). Her TSH level decreased from 76.18 to 3.61 μIU/mL, and fT4 level increased from 63.8 to 154.1 nmol/L (Table 2). The fT4 level was relatively high, but TSH level normalized with no symptoms of hypothyroidism; hence, treatment was terminated after 1 month of herbal medicine administration and 25 sessions of acupuncture treatment. Treatment was well tolerated with no adverse events reported. After 14 months of follow-up, TSH and fT4 levels were maintained.

**Table 2:**
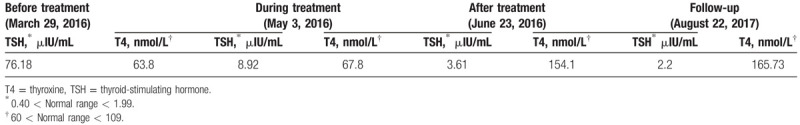
Thyroid function test values of Case 1 before, during and after treatment (s).

### 2.2 Case 2

A 55-year-old woman had experienced coldness of the limbs, as well as shoulder and low back pain, since November 2015. She was originally diagnosed with hyperthyroidism in 2011 and had taken thyroxine suppression therapy for 6 months. She had a history of hysterectomy in 2011. She also had hypersensitivity to coldness, eyelid twitching, and chronic fatigue. Her VAS rating for coldness and back pain was 10.

**Figure 3:**
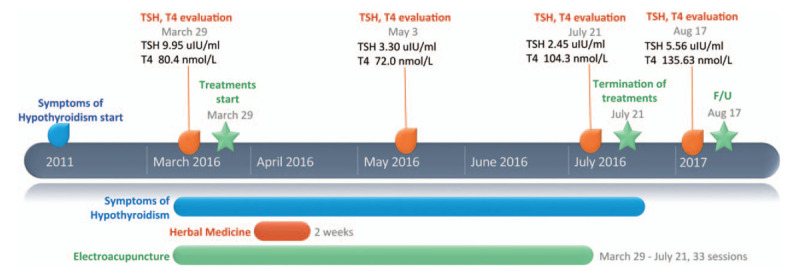
Flow chart depicting treatment in Case 2.

As in Case 1, this patient was diagnosed with ‘Yin deficiency and Yang hyperactivity,’ based on the symptoms of a feeling of heat, and coldness of lower extremities, and diagnosed with primary hypothyroidism based on TSH and fT4 levels (TSH, 9.95 μIU/mL; fT4, 80.4 nmol/L). She received acupuncture and herbal medicine in the same manner as the patient in Case 1 (Table 1 and Fig. 3).

After 4 months of treatment, the patient's fatigue, coldness, and numbness were improved, as all VAS ratings decreased to 3. The patient's TSH decreased from 9.95 to 2.45 μIU/mL, and fT4 increased from 80.4 to 104.3 nmol/L (Table 3).

**Table 3:**
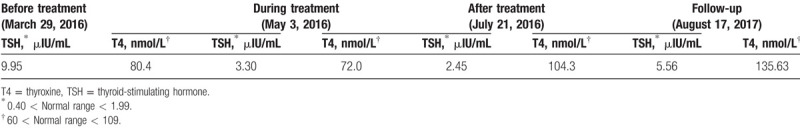
Thyroid function test values of Case 2 before, during and after treatment (s).

Treatment was terminated after 2 weeks of herbal medicine administration and 33 sessions of acupuncture treatment. After 13 months of follow-up, TSH and fT4 levels were maintained in general. After treatment, Case 2 said that she was satisfied with Korean Medicine therapy, and would like to recommend the therapy to other patients. In addition, no evidence of adverse effects was found in the 2 cases.

## 3 Discussion

Hypothyroidism is a common endocrine disorder; however, there are few standardized treatments that are effective for a broad range of patients. Levothyroxine supplement therapy typically requires multiple years of treatment and patients must take the medication during a fasting period (at least 30 minutes before and after meals), often interfering with breakfast. Daily administration over a long period treatment reduces the compliance rate among patients. Additionally, levothyroxine may induce side effects such as acute myocardial infarction, angina pectoris, change in thyroid hormone requirements, or hyporesponsiveness to hormonal therapy.^[17]^

In this report, both patients were administered herbal medicine, without levothyroxine supplementation or additional treatment; both showed marked improvement TSH and fT4 levels. Thyroid function tests revealed stability that was maintained after herbal medicine and acupuncture treatment had been discontinued.

The acupoints treated in these cases relieve hypothyroidism and subsequent symptoms. CV17 and EX2 are used to empower both deficiency of yin-yang; CV12, CV4, and CV6 improve digestive function. LI4, LV3, and ST36 reduce swelling and irrigate the circulatory system in the human body.^[18]^

Herbs were selected to relieve hypothyroid symptoms and modulate thyroid hormone release. *Anemarrhenae Rhizoma*, *Phellodendri Cortex*, and *Glycyrrhizae Radix et Rhizoma* prevent Graves disease and hyperthyroidism;^[19–21]^*Cervi Parvum Cornu* and *Hominis placenta* modulate thyroid hormone production.^[22,23]^ Portions of herbs can be used to manage symptoms of hypothyroidism. *Bupleuri Radix* repairs menstrual disorders,^[24]^ while *Atractylodis Rhizoma Alba* and *Lycii Fructus* exhibit antidiabetic and antiaging effects.^[25,26]^ Nearly all of these herbs can nourish Yin and send pathogenic fire downward.

TSH is a primary barometer for determination of thyroid function.^[27]^ Although both patients exhibited high TSH levels above the upper limit of the normal range at baseline, both patients’ TSH levels were normalized after Korean Medicine treatment. In this case series, this treatment was administered for primary hypothyroidism, but it could be used for patients with menopausal disorder, or partial androgen deficiency of aging males (PADAM) diagnosed with “Yin deficiency and Yang hyperactivity.”

Previous case series reported that Korean medicine using a combined treatment of herbs and acupuncture improved hypothyroidism.^[28,29]^ Herbal medicines, including Leejoong-tang, Bojungikqi-tang, and Saenggangeonbi-tang, were prescribed under similar diagnoses (“Yin deficiency and Yang hyperactivity).” The treatment period ranged from 40 to 184 days. Although the treatment period could be different based on the severity of symptoms, this study had the shortest period of herbal administration.

There were limitations in this case series. We used combination therapies, including a complex formulation of herbs, as well as acupuncture. It is difficult to determine which therapy provided the greatest contribution. Nevertheless, this is the first case series to describe the use of Korean medicine to treat patients with hypothyroidism. Our findings suggest that further studies may be useful to determine the effects of Korean medicine in treatment of thyroid function disorders.

## Acknowledgments

The authors thank the participants and contributors in both cases.

## Author contributions

**Conceptualization:** Sungha Kim, Hyongjun Kim, Jeong-Eun Yoo.

**Data curation:** Sun-Young Moon.

**Funding acquisition:** Jun-Hwan Lee.

**Investigation:** Sungha Kim, Hyongjun Kim, Jeong-Eun Yoo.

**Methodology:** Sungha Kim.

**Resources:** Hyongjun Kim, Jeong-Eun Yoo.

**Supervision:** Sungha Kim.

**Writing – original draft:** Sungha Kim, Hyongjun Kim, Sun-Young Moon.

**Writing – review & editing:** Sungha Kim, Kyungsun Han, Jun-Hwan Lee, Jong Hwan Im, Jeong-Eun Yoo.

Sungha Kim orcid: 0000-0001-5542-3850.

## References

[1] Singer PA Cooper DS Levy EG Treatment guidelines for patients with hyperthyroidism and hypothyroidism. JAMA 1995;273:808–12. 7532241

[2] Garber JR Cobin RH Gharib H Clinical practice guidelines for hypothyroidism in adults: cosponsored by the American Association of Clinical Endocrinologists and the American Thyroid Association. Endocrine Pract 2012;18:988–1028. 10.4158/EP12280.GL 23246686

[3] Vanderpump MPJ The epidemiology of thyroid disease. Br Med Bull 2011;99:39–51. 21893493 10.1093/bmb/ldr030

[4] Unnikrishnan AG Kalra S Sahay RK Prevalence of hypothyroidism in adults: An epidemiological study in eight cities of India. Indian J Endocrinol Metab 2013;17:647–52. 23961480 10.4103/2230-8210.113755 PMC3743364

[5] Seo GH Chung JH Incidence and prevalence of overt hypothyroidism and causative diseases in korea as determined using claims data provided by the Health Insurance Review and Assessment Service. Endocrinol Metab 2015;30:288–96. 10.3803/EnM.2015.30.3.288 PMC4595353 25559717

[6] El-Houni A Younis N Soran H Diarrhoea soon after levothyroxine replacement therapy. QJM 2002;95:125–6. 10.1093/qjmed/95.2.125-a 11861961

[7] Roos A Linn-Rasker SP van Domburg RT The starting dose of levothyroxine in primary hypothyroidism treatment: a prospective, randomized, double-blind trial. Arch Intern Med 2005;165:1714–20. 16087818 10.1001/archinte.165.15.1714

[8] Jonklaas J Bianco AC Bauer AJ Guidelines for the treatment of hypothyroidism: prepared by the american thyroid association task force on thyroid hormone replacement. Thyroid 2014;24:1670–751. 25266247 10.1089/thy.2014.0028 PMC4267409

[9] Wiersinga WM Duntas L Fadeyev V 2012 ETA Guidelines: the use of L-T4 + L-T3 in the treatment of hypothyroidism. Eur Thyroid J 2012;1:55–71. 24782999 10.1159/000339444 PMC3821467

[10] Saravanan P Chau WF Roberts N Psychological well-being in patients on ’adequate’ doses of l-thyroxine: results of a large, controlled community-based questionnaire study. Clin Endocrinol 2002;57:577–85. 10.1046/j.1365-2265.2002.01654.x 12390330

[11] Scavone C Sportiello L Cimmaruta D Medication adherence and the use of new pharmaceutical formulations: the case of levothyroxine. Minerva Endocrinol 2016;41:279–89. 27015567

[12] Arsovska B Zhu J Case report—treating hypothyroidism with acupuncture. Int J Curr Med Pharm Res 2017;3:1556–7.

[13] Woo S-h Kim B-c Shim H-j A case report of cerebral infarction in an elderly patient with subclinical hypothyroidism. J Int Korean Med 2007;28:624–31.

[14] Lee JB Park SW Jeong HY A clinical report of herbal treatment effect on a subclinical hypothyroidism patient. Journal of Haehwa Medicine 2012;21:97–101.

[15] Lianhong L Clinical observation on tonifying spleen and warming the kidney decotion in the treatment of Hashimoto's thyroiditis associated with hypothyroidism. Heilongjiang University of Chinese Medicine 2017.

[16] Chen W-J Zhong M-W Yang J-S 30 cases of Zhen-Wu-Tang added decoction treating primary hypothyroidism categorized by deficiency of yang related to spleen and kidney. New Journal of Traditional Chinese Medicine 2006;3:41–2.

[17] Lee S Park J Lee H Development and validation of Yin-deficiency questionnaire. Am J Chin Med 2007;35:11–20. 17265546 10.1142/S0192415X07004576

[18] Wiersinga WM Thyroid hormone replacement therapy. Horm Res 2001;56: suppl 1: 74–81. 11786691 10.1159/000048140

[19] Zhao Y Wang X Zhao X Acupuncture treatment of 36 cases of hypothyroidism. Shanghai Journal of Acupuncture and Moxibustion 2005;24:25–6.

[20] Xiu L Zhong G Liu D Comparative efficacy and toxicity of different species of Sargassum in Haizao Yuhu Decoction in PTU-induced goiter rats. Evid Based Complement Alternat Med 2017;2017:10. 10.1155/2017/3526186 PMC5497638 28713435

[21] Xu S Zhang F Liu PP Effects of processing on Phellodendri Chinensis Cortex based on material and energy metabolism. Zhong Yao Cai 2015;38:1835–41. 26930977

[22] Yang H Bi X Tang H Clinical efficacy of Yingliu treatment for Graves disease. Int J Clin Exp Med 2015;8:6145–53. 26131218 PMC4483829

[23] Lee D-J Hwangbo M Kwon K Review of cervi cornu parvum pharmacopuncture in Korean medicine. J Pharmacopuncture 2013;16:7–14. 10.3831/KPI.2013.16.008 PMC4331961 25780662

[24] Chae HJ Choi KH Chae SW Placenta Hominis protects osteoporosis in ovariectomized rats. Immunopharmacol Immunotoxicol 2006;28:165–73. 16684675 10.1080/08923970600626197

[25] Zhu L Liang Z-T Yi T Comparison of chemical profiles between the root and aerial parts from three Bupleurum species based on a UHPLC-QTOF-MS metabolomics approach. BMC Complement Altern Med 2017;17:305. 28606186 10.1186/s12906-017-1816-y PMC5468949

[26] Yu M-S Lai CS-W Ho Y-S Characterization of the effects of anti-aging medicine Fructus lycii on beta-amyloid peptide neurotoxicity. Int J Mol Med 2007;20:261–8. 17611646

[27] Sheo H-J Jun S-J Lee M-Y Effects of Lycii fructus extract on experimentally induced liver damage and alloxan diabetes in rabbits. J Korean Soc Food Sci Nutr 1986;15:136–43.

[28] Castellano C-A Laurin D Langlois M-F Thyroid function and cognition in the euthyroid elderly: a case-control study embedded in Quebec longitudinal study. Psychoneuroendocrinology 2013;38:1772–6. 23507188 10.1016/j.psyneuen.2013.02.013

[29] Lim B Kim C Park J A review of domestic clinical studies about clinical and subclinical hypothyroidism treated with Korean medical interventions. J Intern Korean Med 2018;39:645–61.

[30] Lee M Choi Y Systemic reviews of domestic experimental studies of herbal medicines used for hypothyroidism since 2000. J Intern Korean Med 2015;36:570–81.

